# Conserved thermal performance curves across the geographic range of a gametophytic fern

**DOI:** 10.1093/aobpla/ply050

**Published:** 2018-09-12

**Authors:** Sally M Chambers, Nancy C Emery

**Affiliations:** 1 Department of Botany and Plant Pathology, Purdue University, West Lafayette, IN, USA; 2 Department of Ecology and Evolutionary Biology, University of Colorado, Boulder, CO, USA

**Keywords:** Climate change, ferns, gametophyte, geographic range, manipulative experiment, population differentiation, temperature, thermal performance curve, *Vittaria appalachiana*

## Abstract

Species-level responses to environmental change depend on the collective responses of their constituent populations and the degree to which populations are specialized to local conditions. Manipulative experiments in common-garden settings make it possible to test for population variation in species’ responses to specific climate variables, including those projected to shift as the climate changes in the future. While this approach is being applied to a variety of plant taxa to evaluate their responses to climate change, these studies are heavily biased towards seed-bearing plant species. Given several unique morphological and physiological traits, fern species may exhibit very different responses from angiosperms and gymnosperms. Here, we tested the hypothesis that previously detected population differentiation in a fern species is due to differentiation in thermal performance curves among populations. We collected explants from six populations spanning the species’ geographic range and exposed them to 10 temperature treatments. Explant survival, lifespan and the change in photosynthetic area were analysed as a function of temperature, source population and their interaction. Overall results indicated that explants performed better at the lowest temperature examined, and the threshold for explant performance reflects maximum temperatures likely to be experienced in the field. Surprisingly, explant fitness did not differ among source populations, suggesting that temperature is not the driver behind previously detected patterns of population differentiation. These results highlight the importance of other environmental axes in driving population differentiation across a species range, and suggest that the perennial life history strategy, asexual mating system and limited dispersal potential of *Vittaria appalachiana* may restrict the rise and differentiation of adaptive genetic variation in thermal performance traits among populations.

## Teaser

Thermal tolerance limits in ferns remain poorly understood, and even less is known about these limits at the population level. Being the second most diverse group of vascular land plants, this leaves a large gap in our understanding of how contemporary plant species will respond to elevated temperatures that will be occurring in the future. This study, focusing on the interesting species *Vittaria appalachiana*, indicated that thermal limits across all populations are relatively conserved and correspond highly to those experienced in the field, leaving little room for adaptation and tolerance in light of elevated temperatures in the future.

## Introduction

Species’ responses to the environmental variation throughout their geographic ranges depend on the collective tolerances of the constituent populations. The extent to which populations evolve different tolerances is expected to depend on the spatial scale of gene flow relative to the grain of environmental heterogeneity (Van Tienderen 1991; Sultan and Spencer 2002). High rates of gene flow among populations experiencing different selective pressures should favour the evolution of phenotypic plasticity, while asexual reproduction and restricted dispersal will favour specialization to local conditions (Bradshaw 1965; Kawecki and Ebert 2004; Sherman and Ayre 2008; but see Gray and Goddard 2012; Sexton *et al.* 2014). Gene flow among populations can be heavily influenced by the spatial distribution of habitat across a species’ range, as populations that are restricted to patchy or fragmented habitats will experience less interpopulation gene flow (Primack and Miao 1992; Thomas 2000). Furthermore, over evolutionary time, patchy habitat structure itself may generate selection for localized dispersal strategies due to the fitness consequences of dispersing propagules that land in unsuitable habitat between patches, generating a feedback between the evolution of environmental specialization and localized dispersal (Cody and Overton 1996; Cheptou *et al.* 2008; Schenk 2013; Van Den Elzen *et al.* 2016). In species where populations are locally specialized, the species’ geographic range as a whole may reflect a broader range of environmental conditions than each individual population can tolerate.

Population-specific responses to environmental gradients can be examined using a variety of lab and field experiments (Lawlor and Mitchell 1991; Norby *et al.* 1999; Charles and Dukes 2009; Marsico and Hellmann 2009; Samis and Eckert 2009; Agren and Schemske 2012; Chambers and Emery 2016). Common garden experiments that include experimental manipulations have proven to be a powerful tool for isolating the effects of specific environmental variables (such as temperature) that are hypothesized to drive population-level differences in performance (Marion *et al.* 1997; McLeod and Long 1999; Grime *et al.* 2000) and the potential for populations to tolerate conditions that do not presently occur in their local environments. Reaction norms are a particularly useful way to quantify the effects of an environmental factor (e.g. temperature) on the phenotype (e.g. plant size) of genotypes from different populations. Reaction norms that represent fitness across temperature gradients, often called thermal performance curves (Kingsolver *et al.* 2004; Gilchrist 2015), can be compared among genotypes using a variety of techniques, ranging from relatively straightforward comparisons of the slopes and intercepts of the lines that represent phenotypic responses across two different environments (Bradshaw 1965, 1972; Schmitt 1993; Dorn *et al.* 2000), to more complex approaches that consider the shape of reaction norm curves across three or more environmental levels (Izem and Kingsolver 2005; Stinchcombe *et al.* 2012; Murren *et al.* 2014). Quantifying fitness and phenotypic responses of multiple populations across multiple levels of an environmental axis makes it possible to test for microevolutionary divergence among populations (Murren *et al.* 2014).

To date, the majority of experimental research that has evaluated plant responses to climate change has focused on seed-bearing plants, with comparatively less attention directed towards other major land plant lineages (Gignac 2001; Page 2002). Ferns are the second most diverse group of vascular plants on the planet, yet their ecology is severely understudied in comparison to seed-bearing plants. Ferns play significant ecological roles in their communities (George and Bazzaz 1999; Amatangelo and Vitousek 2008) and are often considered to be indicators of habitat quality and environmental change (Page 2002; Bassler *et al.* 2010; Bergeron and Pellerin 2014). Furthermore, a number of unique life history and physiological traits may cause ferns to exhibit different responses to climate change compared to angiosperms and gymnosperms (see Banks 1999 for a thorough review). One important difference between ferns and angiosperms is that both the gametophyte and the sporophyte are free-living and independent in ferns, while the gametophyte is highly reduced and dependent on the sporophyte in angiosperms and gymnosperms. Most fern gametophytes are one cell-layer thick, photosynthetic, lack cuticle or stomata and produce antheridia, archegonia and rhizoids, and thus are physiologically quite different from their sporophyte counterparts in ways that may have significant consequences for their responses to temperature variation. Despite their relatively small size and often delicate appearance, fern gametophytes are often more robust to environmental extremes than their respective sporophytes (Farrar 1978; Sato and Sakai 1981; Watkins *et al.* 2007; Pinson *et al.* 2017). Given the fact that fertilization, and even asexual reproduction in some species, occurs during the gametophyte portion of the life cycle, understanding how fern gametophytes respond to different temperatures is important for predicting the overall effects of climate change on fern lineages.

Throughout the Appalachian Mountains and Appalachian Plateau of eastern North America are a number of recesses in rock outcroppings called ‘rockhouses’ or ‘rockshelters’. These formations developed from the weathering of soft bedrock located below layers of sandstone, generating large sandstone overhangs. The recesses below these overhangs support a diverse flora (Walck *et al.* 1996,^1997^; Oberle and Schaal 2011), including a variety of fern species, some of which are endemic to these unique habitats (Farrar 1967, 1998; Watkins and Farrar 2002, 2005; Testo and Watkins 2011). A handful of these endemics are temperate fern species that are phylogenetically nested within tropical clades that may have retreated to the buffered thermal environments inside rockshelters during past glaciation events (Farrar 1990; Walck *et al.* 1996; Chambers and Emery 2016; Pinson and Schuettpelz 2016). *Vittaria appalachiana* is one of the few species endemic to these rockshelters that never produces a viable sporophyte, but rather reproduces only asexually via gemmae and vegetative spread (Farrar 1967, 1978, 2016). Populations of *V. appalachiana* occupy rockshelters from northern Alabama to south-western New York, spanning a total of 9° in latitude (Farrar and Mickel 1991). While rockshelters can buffer these populations from fluctuations in temperature (Farrar 1998; Chambers and Emery 2016), the latitudinal distribution exposes populations to different average thermal conditions (Table 1).

**Table 1.** Source population locality information and summary temperature data for each site. Temperature data were collected within populations at each site between 2010 and 2013 (Chambers and Emery 2016). Temperature treatments used in the experiment spanned the range of average temperatures experienced by natural populations in the field as well as higher temperatures that are expected by 2100 under climate change projections.

Population	Range location	Coordinates		Daily average (°C)	Daily minimum (°C)	Daily maximum (°C)
Cane Creek Nature Preserve (Colbert Co., AL)	Southern	34 37.27N	87 47.88W	15.38	14.06	16.77
Jones Property (Transylvania Co., NC)	Eastern	35 11.44N	82 42.88W	12.40	11.08	14.17
Pennyrile State Park (Christian Co., KY)	Western	37 04.54N	87 39.95W	14.77	13.61	15.99
Hemlock Cliffs (Crawford Co., IN)	Central	38 16.38N	86 32.20W	–	–	–
Deep Woods (Hocking Co., OH)	Central	39 24.49N	82 34.60W	11.51	10.20	12.78
Rock City Park (Cattaraugus Co., NY)	Northern	42 04.79N	78 28.62W	7.74	6.92	8.70
Temperature grand average				11.97	10.81	13.25

Given the temperature variation encompassed within the species’ range, and results of previous studies documenting dispersal limitation (Stevens and Emery 2015), and population differentiation (Chambers and Emery 2016; Chambers *et al.* 2017) among *V. appalachiana* populations, we predicted that *V. appalachiana* would exhibit population differentiation in their responses to a thermal gradient (i.e. their thermal tolerance curves). We also predicted that all *V. appalachiana* populations would have a relatively narrow range of temperature tolerances along a temperature gradient, as *V. appalachiana* is restricted to relatively climatically buffered microhabitats and therefore would not have experienced selection to tolerate a broad range of temperature conditions.

## Methods

### Sample collection

We identified six populations from different locations across the geographic range of *V. appalachiana* using occurrence information obtained from non-profit organizations (New York Natural Heritage Program and North Carolina Natural Heritage Program), state botanists and previously published locality data (Farrar and Mickel 1991; Table 1). Populations included in this study were selected based on site accessibility and our ability to secure permission to collect samples from the resident populations. In October of 2012, 30 gametophyte explants were collected from each of the six source populations over an 8-day period (*N* = 180). Based on the results of genetic studies that evaluated the distribution of genetic variation within and among populations of *V. appalachiana* (Farrar 1990), it is most likely that explants from a single site represented replicates of the same vegetative clone, but that clones differed between sites.

Within each rockshelter, samples were collected from random positions along a horizontal transect that spanned the length of each population. A blade was used to carefully detach gametophytes that were directly attached to rock surfaces or sandy substrates within rockshelters. After removal, each sample was trimmed to a standardized circle with a 4.5-mm diameter (which contained roughly 10–20 individual thalli), and placed on an agar medium containing half-strength Murashige and Skoog Basal Salt Mixture (Sigma, St. Louis, MO, USA) supplemented with 0.5 mL/L plant vitamins, 1.0 mL/L Benomyl, titrated to a pH of 6.5 using potassium hydroxide, and solidified with 0.65 % agar (Sigma, St. Louis, MO, USA).

After samples were collected from all populations, each explant was transferred to a fresh agar plate to minimize growth of contaminants. The samples were then placed in a Revco RI-50-555-A growth chamber at 20 °C, under 0.8 μmol light levels following an 8-h light:16-h dark cycle, for seven months to establish on the agar medium and recover from any stressors associated with collection. Field measurements indicated that populations experience light levels between 0 and 5.99 μmol m^−2^ s^−1^, averaging 0.5 μmol m^−2^ s^−1^, and temperatures between −3.70 and 27.60 °C (S. M. Chambers, unpubl. data); thus, the growth chamber conditions in which explants were maintained fell within the conditions they experience in their natural habitat.

### Temperature treatments

**Figure 1.**
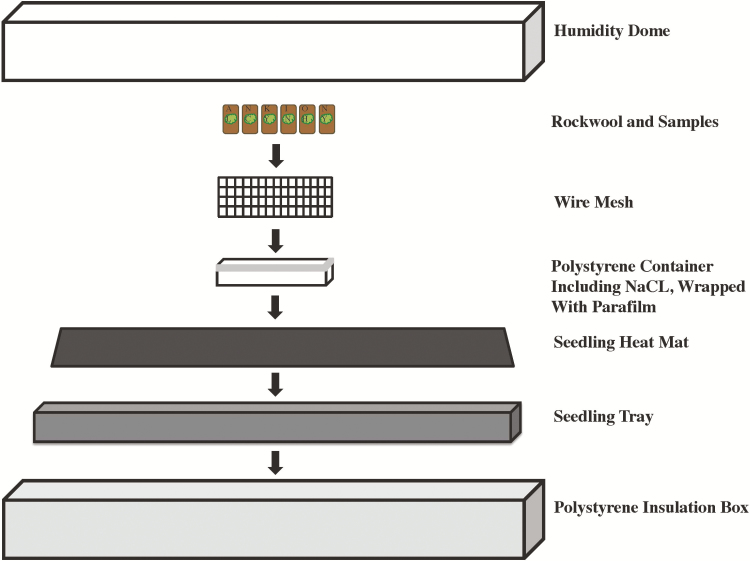
Schematic drawing of the experimental design for treatments in which temperatures exceeded 20 °C. *Vittaria* explants were placed on rockwool and arranged on a wire mesh tray. The explants and the wire mesh were placed together in a clear, plastic, polystyrene container that contained a sodium chloride salt solution to maintain a consistent humidity level. Each polystyrene box was placed on top of a seedling heat mat (Hydrofarm, Inc., Petaluma, CA, USA) that was programmed to a specified temperature, set in a plastic seedling tray and covered with a humidity dome (not depicted in this schematic). Each seedling tray, consisting of one temperature treatment, was placed in a handmade polystyrene box to insulate the seedling tray to maintain constant temperature conditions. Because the minimum temperature setting for the seedling heat mats was 20 °C, temperature treatments of 9, 12, 15 and 18 °C were generated by placing a heat mat set to 20 °C outside of the seedling tray and elevating the trays 5, 3, 1 and/or 0 cm, respectively, above a heat mat set to 20 °C. The lowest temperature treatment, 6 °C, was imposed by placing the tray on a heat mat that was turned off.

After transplants had established, we exposed each explant to 1 of 10 different temperature treatments and measured its response over 22 weeks. Temperatures were selected to span the average and maximum temperatures that we had previously measured in each of the sampled populations over a 3-year period prior to this experiment (6–18 °C, measured between 2010 and 2013), as well as the elevated temperature levels projected to occur by the end of the 21st century due to global climate change (21–30 °C; IPCC 2013; Table 1). Specific temperature level treatments were 6, 9, 12, 15, 18, 21, 24, 26, 27 and 30 °C. The entire temperature gradient was replicated in three different growth chambers. Temperature was manipulated within each chamber placing explants from each population inside heated and insulated containers that increased temperature above the base growth chamber temperature of 6 °C using seedling heating mats (Fig. 1). One explant from every population was exposed to each temperature treatment in each growth chamber, resulting in three replicates of the entire temperature gradient per population.

In May of 2013, each explant was transferred from the agar medium to a 2.5 × 1.25 × 0.5 cm section of rockwool, a suitable substrate for propagating *V. appalachiana* because it resembles the porous sandstone that is the natural substrate for most *V. appalachiana* populations. Prior to transfer, the rockwool was moistened with a liquid nutrient medium (created as above without agar; see ‘Sample collection’) to facilitate establishment. A pilot experiment indicated that explant performance was greatest when we minimized the fluctuations in relative humidity experienced each time a container was opened to collect data. Consequently, we standardized the relative humidity for all explants to 75 %, which was the level maintained in the laboratory during the course of the experiment, by placing explants on wire trays over a sodium chloride (NaCl) salt solution inside their container (Fig. 1). The 75 % humidity level is slightly lower than levels we have measured in the field during daylight hours in the summer months, which ranged between 85 and 95 %, but are likely within levels experienced over the course of daily and annual temperature cycles (S. M. Chambers, unpubl. data).

One explant from each population was placed in each polystyrene container that was wrapped with parafilm and placed on a seedling heating mat inside a seedling tray. Each seedling tray was placed inside a large insulated polystyrene box and covered with a clear humidity dome to help maintain temperature and humidity levels within each treatment (Fig. 1). Sodium chloride salt solutions were replaced every 2 weeks to ensure relative humidity was kept at a constant level. To prevent explant dehydration, 500 μL of deionized water was added directly to the rockwool substrate every 2 weeks for the duration of the experiment.

We measured explant fitness as (i) explant survival (binary yes/no), (ii) lifespan (days) and (iii) changes in the area of visible photosynthetically active tissue. Given that *V. appalachiana* only reproduces asexually via the production of gemmae along the margins of the gametophyte thalli, the length of time an explant survives and thalli surface area together provide an estimate of the number of gemmae it produces and therefore serve as proxies for fitness. Explant survival and lifespan were monitored every 2–3 days for the first 6 weeks of the experiment, and once per week for the final 16 weeks. During each census date (i.e. the dates when data were collected), an explant was recorded as ‘alive’ if any photosynthetically active (green) tissue was visible to the naked eye. The amount of surface area occupied by photosynthetically active tissue was determined from digital photographs that were taken of each explant at the midpoint (week 9) and end (week 22) of the experiment, thus capturing the change in photosynthetic area (PA) over two consecutive time periods. Photographs were taken with a Cannon PowerShot® SX130IS placed on a ProMaster® 7050 tripod to ensure a consistent pixel ratio among images. Using these photographs, we calculated PA by outlining photosynthetic tissue using GIMP 2.0 (Peck 2008; Solomon 2009), and calculating the area using ImageJ (Rasband 1997; Schneider *et al.* 2012). Changes in PA during these two time periods were calculated for each surviving explant by dividing the difference in PA (current PA − initial PA) by the initial PA, where the initial PA was the PA measured at the beginning of the experiment. Changes in PA were calculated for each explant at the midpoint and end of the experiment.

### Statistical analyses

#### Survival.

We tested if variation in survival of *V. appalachiana* experimental explants was explained by population identity, temperature treatment and the interaction between population and temperature using a generalized linear mixed model (PROC GLIMMIX; SAS v. 9.4) with a logit link function to account for the binary response variable (Liang and Zeger 1986; Ziegler *et al.* 1998). Temperature was treated as a continuous predictor variable, and source population was treated as a fixed categorical predictor variable. The temperature × treatment interaction was also included in the model. We applied a spatial power covariance structure (*sppow*), specifying ‘census date’ as the spatial factor and the interaction between source population, temperature and growth chamber was identified as the ‘subject’ statement in the model. A separate random statement was also included to specify that growth chamber was a random factor. Census date was included in the ‘random’ statement to account for repeated measurements.

#### Lifespan.

The length of time that explants survived under the different temperature conditions was evaluated in a mixed-model ANCOVA (PROC MIXED; SAS v. 9.4). Lifespan (i.e. the number of days that an explant survived under experimental conditions) was evaluated as a function of population identity (considered a categorical variable) and temperature (considered a continuous variable). The population × temperature interaction was included in the analysis, and growth chamber was included as a random effect.

#### Changes in PA.

The change in PA ((current PA − initial PA)/initial PA) was analysed using a mixed-model repeated-measures ANOVA (PROC MIXED; SAS v. 9.4). We specified an autoregressive covariance structure with source population, temperature and time period (i.e. first or second half of the experiment) as fixed main effects. Growth chamber was treated as a random factor, and a repeated statement was used to account for the two estimates of the change in PA per explant (one at the midpoint of the experiment, and one at the end). We included all two- and three-way interactions among population, temperature and time period in the model. We included time period as a main effect to test if the change in PA differed significantly between the first and second time period of the experiment, while simultaneously accounting for the non-independence of repeated measures on the same explant. Tukey tests were used to conduct pairwise comparisons of statistically significant main effects.

#### Pairwise comparisons of thermal performance curves.

We evaluated population differences in thermal response curves by calculating the *offset*, *slope*, *curvature*, *wiggle* and *total* parameters for continuous reaction norms as defined by Murren *et al.* (2014). Four of these metrics (*offset*, *slope*, *curvature* and *wiggle*) partition the total variation that exists between a pair of thermal performance curves (TPCs) and the *total* metric represents the sum of those differences. For all metrics, smaller values indicate greater similarity. The metrics *offset* and *slope* are the most similar to traditional linear models and comparisons of performance curves, while *curvature* and *wiggle* represent higher-order statistical comparisons between performance curves. Therefore, estimates of *slope* and *wiggle* for population pairs may reveal microevolutionary variation in reaction norms that are otherwise obscured using traditional linear analyses (Murren *et al.* 2014). Each metric was calculated separately for each measurement of explant performance (survival, lifespan, changes in PA), as follows:

The *offset* represents the average difference in performance between a pair of populations in any treatment, and is calculated as:

$$Offset=\;\left|\frac{\sum_{\text{1}}^{n}D_{i}}{n}\right|$$

where *D* represents the difference in mean performance in treatment *i*, and *n* is the total number of treatments (here, *n =* 10).

The *slope* parameter calculates the average change in *D* from treatment *i* to *i +* 1 (e.g. between 6 and 9 °C):

$$Slope=\left|\frac{\sum_{\text{1}}^{n-\text{1}}S_{i}}{n-\text{1}}\right|$$

where *S*_*i*_ = *D*_*i+*1_ – *D*_*i*_.

*Curvature* is the average change in slope across treatments:

$$Curvature=C=\left|\frac{\sum_{\text{1}}^{n-\text{2}}C_{i}}{n-\text{2}}\right|$$

where *C*_*i*_ = *S*_*i+*1_ – *S*_*i*_.

*Wiggle* captures any remaining variation in the comparison of two performance curves, and is calculated as the sum of the absolute value of the change in slope after removing the estimate of curvature:

$$Wiggle=\frac{\sum_{1}^{n-2}\left|C_{i}\right|}{n-2}-C$$

A final metric, *Total*, summarizes the cumulative differences between two tolerance curves, as estimated by the other four metrics:

Total=O+S+C+W

To facilitate comparisons among population pairs that varied in overall performance, each metric was standardized by dividing the estimated value for *offset*, *slope*, *curvature*, *wiggle* and *total* by the grand mean performance measure of both populations in each pairwise comparison. Calculations for these equations were conducted using the base package in R (R Core Team 2014).

## Results

### Survival

**Figure 2.**
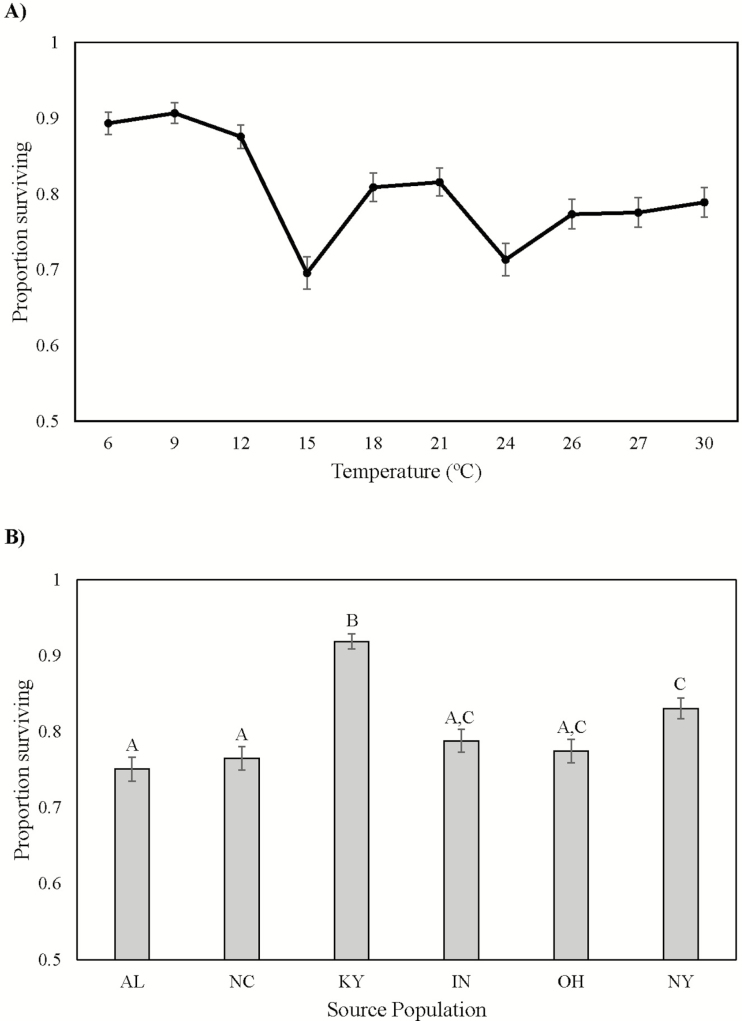
(A) The proportion of individuals surviving in each temperature treatment averaged across all source populations and census dates. Data represent raw means ± 1 SE. (B) The proportion of individuals from each population surviving, averaged across all temperature treatments and census dates. Data shown are raw means ± 1 SE.

Survival rates differed significantly across temperature treatments (Temperature, Table 2; Fig. 2A), among populations (Population, Table 2; Fig. 2B), and with respect to populations within each temperature treatment (Temperature * Population, Table 2). Overall survivorship was highest at temperatures below 15 °C and lowest at 15 and 24 °C (Fig. 2A). Explants from Kentucky (KY) had significantly higher survivorship than all other populations followed by New York (NY), which had significantly higher survivorship than Alabama (AL) and North Carolina (NC) (Fig. 2B).

**Table 2.** Statistical results of all analyses evaluating explant performance from six different populations across 10 temperature treatments. The ‘test statistic value’ column reports *F* statistics for the main effects in the survival, lifespan and PA analyses. For the latter two analyses, test statistic values for growth chamber are reported as *Z* statistics because this factor was a random effect. Subscripts identify the numerator and denominator degrees of freedom where appropriate. *P*-values that were statistically significant are indicated with bold text.

Factor	Test statistic value	*P*-value
Survival		
Temperature	49.10_1, 4488_	**0.0004**
Population	4.52_5, 4488_	**<0.0001**
Temperature * Population	3.10_5, 4488_	**0.0086**
Lifespan (days)		
Temperature	17.80_1, 166_	**<0.0001**
Population	0.77_5, 166_	0.5749
Growth chamber	0.72_2_	0.2353
Temperature * Population	0.70_5, 166_	0.6250
PA		
Temperature	31.61_1, 330_	**<0.0001**
Population	3.14_5, 330_	**0.0087**
Time period	94.80_1, 330_	**<0.0001**
Growth chamber	0.79_2_	0.2147
Temperature * Population	1.77_5, 330_	0.1178
Temperature * Time period	29.06_1, 330_	**<0.0001**
Population * Time period	1.77_5, 330_	0.1177
Temperature * Population * Time period	0.88_5, 330_	0.4978

### Lifespan

**Figure 3.**
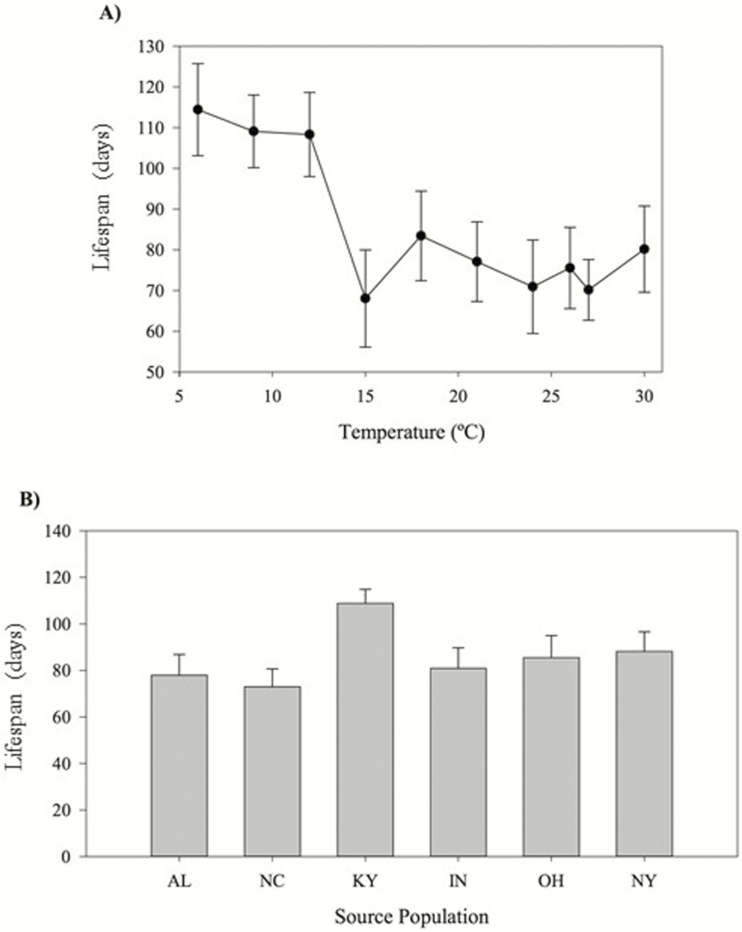
(A) The lifespan, or number of days that explants remained alive in each temperature treatment, averaged across all populations (raw means ± 1 SE). (B) Mean lifespan for explants from each source population, averaged across all temperature treatments. Data shown are raw means ± 1 SE.

The overall mean lifespan of explants during the experiment was ~86 days. Explants exposed to temperatures of 15 °C or higher survived roughly a month less than explants grown at cooler temperatures (Fig. 3A), leading to an overall significant effect of temperature on lifespan (Temperature, Table 2). The length of time that explants remained alive did not vary significantly among populations overall (Population, Table 2; Fig. 3B), and the effects of the temperature treatments were relatively consistent among populations (see non-significant Temperature × Population interaction, Table 2).

### Changes in PA

**Figure 4.**
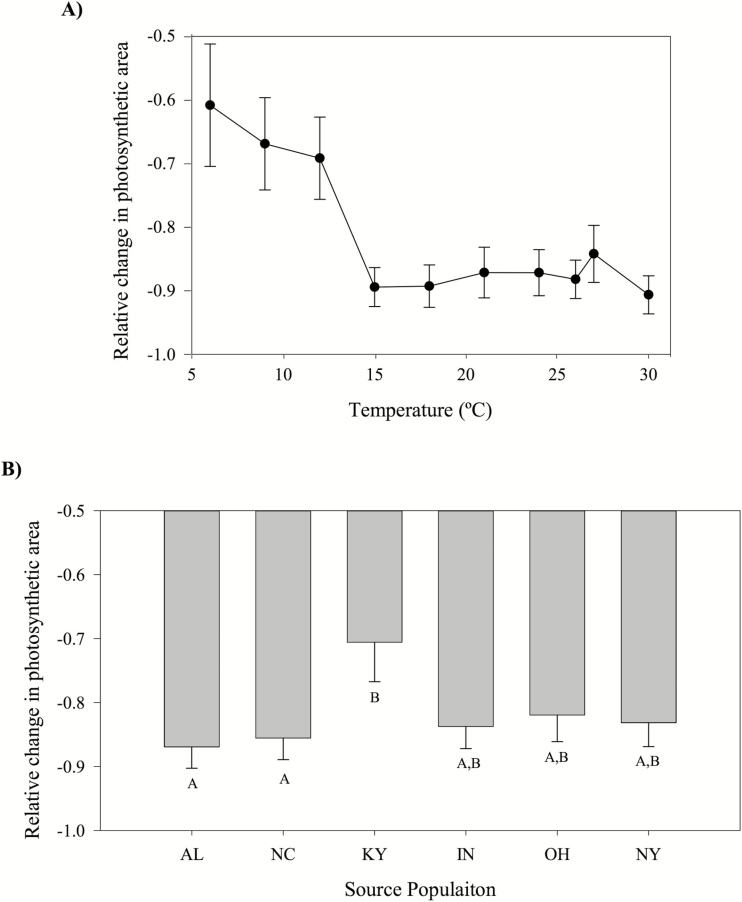
(A) Mean change in PA (current PA − initial PA/initial PA) for each temperature treatment averaged across all humidity treatments and source populations (raw means ± 1 SE). (B) Mean change in PA for each source population, averaged across all temperature treatments (raw means ± 1 SE). In all panels, a smaller reduction in PA indicates a larger amount of initial PA remaining. In both panels, values around −0.5 indicate that nearly half of the PA was lost, while those closer to −1 indicate nearly all of the PA was lost.

We observed a significant increase in the rate of decline in explant PA with increasing temperature (Table 2; Fig. 4A), and the total reduction in PA varied significantly among source populations (Population, Table 2), with Kentucky (KY) retaining the greatest amount of PA and Alabama (AL) losing the most, on average, over all temperature treatments and the two time periods (Fig. 4B). The effect of temperature varied significantly between the first and second time periods (Temperature × Time period interaction, Table 2), and explants lost PA faster in the first time period (Time period, Table 2). The change in PA for all remaining factors and interactions in the model were not statistically significant (Table 2).

### Pairwise comparisons of thermal performance curves

Overall patterns in the cumulative differences between performance curves (*total*) indicated that Indiana (IN) and North Carolina (NC) had the most similar TPCs while New York (NY) and Indiana (IN) exhibited the greatest differences in all three measures of explant performance **[see****Supporting Information—Table S1a**–**c****]**. Additional differences among populations emerged when the four Murren metrics—*offset*, *slope*, *curvature* and *wiggle*—were individually examined. The mean difference in explant performance (*offset*) for all three explant performance metrics was greatest between explants from New York (NY) and Indiana (IN) **[see****Supporting Information—Table S1a**–**c****]**. On the other hand, the average change in mean performance between temperature steps (*slope*) was greatest between Ohio (OH) and Alabama (AL) for survival, Ohio (OH) and Indiana (IN) for lifespan, and New York (NY) and Indiana (IN) for change in PA **[see****Supporting Information—Table S1a**–**c****]**. We detected a relatively large degree of dissimilarity between Alabama (AL) and Indiana (IN) in *curvature* and *wiggle* when lifespan was used as the measure of performance **[see****Supporting Information—Table S1b****]**, but no clear patterns emerged for these two metrics when examining survival and change in PA **[see****Supporting Information—Table S1a****and****c****]**.

## Discussion

Our results indicate that patterns of population differentiation in *V. appalachiana* that had been previously documented in the field and physiological studies (Chambers and Emery 2016; Chambers *et al.* 2017) are not driven by population variation in temperature tolerances. These previous studies detected a pattern of countergradient variation (Conover and Schultz 1995) in *V. appalachiana* in which explants from New York outperformed all other populations over much of the species’ geographic range. However, the results from this experiment suggest that in general populations have very similar temperature tolerance curves, with explants from Kentucky (KY) having higher survivorship and PA retention than several other populations (Figs 2B and 4B). The Murren metrics identified more subtle differences between specific population pairs that were not evident in the linear analyses, though the nature of these differences depended on the performance metric that is analysed. For example, estimates of *curvature* and *wiggle* detected the greatest difference between explants from Alabama and Indiana with respect to lifespan, suggesting that there may be slight differences in the effects of temperature variation on explant longevity between these two populations **[see****Supporting Information—Table S1b****]**. Nonetheless, only faint patterns of population differentiation in thermal performance emerged in *V. appalachiana* using both traditional linear analyses and higher order comparisons, suggesting that TPCs are relatively conserved within this species even though its populations span a relatively broad latitudinal range in North America.

The significant differences we detected among populations in the TPCs for explant survival and change in PA appeared to be driven by two populations, Kentucky (KY) and New York (NY). All other populations exhibited similar TPCs across the temperature gradient tested here, suggesting that temperature variation has not driven the patterns of population differentiation previously detected in field transplant experiments (Stevens and Emery 2015; Chambers and Emery 2016). It is possible that the previously detected patterns of population differentiation have been driven by factors other than mean temperature that are known to vary across the species’ range, such as relative humidity levels (Chambers *et al.* 2017). Responses to dry conditions share a metabolic pathway with responses to cold temperatures, such that organisms that can tolerate colder temperatures can also tolerate lower levels of relative humidity (Knight and Knight 2001; Sinclair *et al.* 2013). Therefore, if we had examined population responses to low temperatures, perhaps we would have identified the same pattern of differentiation that had been documented in field studies. While the high overall levels of senescence that we observed could generate concern that the experimental environment was unusually stressful for the explants, these levels of senescence are actually relatively typical for this species. A previously conducted reciprocal transplant experiment indicated similar patterns of senescence among gametophytes transplanted back in to their home environment (Chambers and Emery 2016). Additional work conducted some 40 years ago also comments on the overall slow growth rate (Farrar 1978). Thus, the senescence rates observed here are not far from the norm with respect to experiments in this species.

It is quite possible that the biogeography and history of selection in *V. appalachiana* can largely explain the surprisingly similar thermal responses we observed among widely distributed populations. *Vittaria appalachiana* was likely once widespread in eastern North America, fully capable of producing a sporophyte when the climate resembled that of the contemporary neotropics prior to the Pleistocene glaciations (Farrar 1998). It is thought that *V. appalachiana* retreated into the rockshelters at that time to escape the cold climates of the Pleistocene glacial period. Selection during this time period may also have favoured the obligate gametophyte life history strategy because the sporophyte may have been less tolerant of cold temperatures (Farrar 1998). These pressures would have been imposed on all populations, and the loss of the sporophyte generation and highly fragmented distribution left these populations with no potential for sexual reproduction and little potential for gene flow. As a result, there is little opportunity for adaptive genetic variation to arise in these populations, highly restricting their potential for adaptive differentiation. Furthermore, the buffered climate within rocksehlters may limit the extent to which populations experience temperature variation occurs across their geographic range. Under this biogeographic hypothesis, differences observed in the previous field studies are more likely due to genetic drift among populations that established in the Pleistocene rather than patterns of local adaptation to contemporary environmental conditions.

While our results did not find strong evidence for adaptive differentiation among populations in their TPCs, we certainly observed that the species as a whole is highly sensitive to the temperature gradient that was experimentally imposed. All populations exhibited a decline in performance in temperatures above 12 °C for all performance metrics (Figs 2A, 3A and 4A). Previous studies have found that 15 and 18 °C are the highest average and maximum temperatures known to occur in natural populations (Table 1; Chambers and Emery 2016); thus, 12–15 °C may represent a critical species-wide temperature threshold for *V. appalachiana*. The experimental temperatures that represented climate change scenarios (i.e. 21, 24, 26, 27 and 30 °C) resulted in rapid explant deterioration and senescence. The clear negative effects of temperatures above the 15 °C threshold for all populations indicate that many, if not all, populations of *V. appalachiana* will be pushed beyond their physiological tolerance limits as temperature rapidly increases due to global climate change (IPCC 2013). Transplant experiments beyond *V. appalachiana*’s northern range boundary have shown that suitable habitat exists at higher latitudes but has not been colonized due to the highly limited dispersal potential of the species (Stevens and Emery 2015). Just as the lack of sexual reproduction and inability to disperse may have restricted the potential for populations to adapt to their local temperature regimes, these characteristics will also likely restrict the potential for rapid adaptive evolution in response to changing thermal environments. Management practices that aim to conserve this species, and others that are dispersal limited, asexual and physiologically sensitive to climate, may be required to use assisted migration techniques, which would facilitate the colonization of suitable habitat that becomes available as climate change unfolds.

## Conclusions

The results of this study indicate that the thermal performance curves of *V. appalachiana* populations are highly conserved despite a relatively broad latitudinal range occupied by the species. This suggests that adaptive variation may not arise and spread in taxa with limited potential for gene flow within and among populations. Our results also highlight the importance of examining different aspects of reaction norm shapes to uncover differences not captured by traditional reaction norm comparisons.

## Sources of Funding

This work was supported by a Purdue Andrews Environmental Travel Grant and the Botany and Plant Pathology Department at Purdue University.

## Contributions by the Authors

S.M.C. and N.C.E. conceived and designed the experiment. S.M.C. conducted the experiment, and analysed the data. S.M.C. and N.C.E. wrote the manuscript.

## Conflict of Interest

None declared.

## Supplementary Material

SupplementaryInformation

## Supporting Information

The following additional information is available in the online version of this article—

Table S1a–c. Pairwise values for all five Murren metrics, for all three measurements of explant performance.
