# Supplementary material for: Conserved thermal performance curves across the geographic range of a gametophytic fern
Source: AoB Plants. 2018 Sep 12;10(5):ply050. doi: 10.1093/aobpla/ply050 (PMC6185718; doi:10.1093/aobpla/ply050)
Supplement: SupplementaryInformation [file ply050_suppl_supplementary_information.docx]

**Table S1.** Pairwise population comparisons of thermal performance curves following the methods for comparing continuous reaction norms in Murren et al. (2014). Each panel represents one metric of explant performance (survival, time to death, and change in PA), and the values in each cell indicate the pairwise differences among populations in their thermal performance curves as calculated by the five Murren metrics (*total*, *offset*, *slope*, *curvature*, and *wiggle*). The largest and smallest values for each reaction norm and explant performance metric are indicated in bold.

**a) Survival**

|  | **Total** | | | | | |
| --- | --- | --- | --- | --- | --- | --- |
|  | AL | NC | KY | IN | OH | NY |
| AL | – | – | – | – | – | – |
| NC | 0.154 | – | – | – | – | – |
| KY | 0.309 | 0.339 | – | – | – | – |
| IN | 0.152 | **0.104** | 0.248 | – | – | – |
| OH | 0.183 | 0.216 | 0.211 | 0.573 | – | – |
| NY | 0.224 | 0.158 | 0.190 | **0.783** | 0.192 | – |

**Table S1a (cont’d)**

|  | **Offset** | | | | | | **Slope** | | | | | |
| --- | --- | --- | --- | --- | --- | --- | --- | --- | --- | --- | --- | --- |
|  | AL | NC | KY | IN | OH | NY | AL | NC | KY | IN | OH | NY |
| AL | – | – | – | – | – | – | – | – | – | – | – | – |
| NC | **0.014** | – | – | – | – | – | 0.040 | – | – | – | – | – |
| KY | 0.218 | 0.204 | – | – | – | – | 0.067 | 0.031 | – | – | – | – |
| IN | 0.049 | 0.035 | 0.170 | – | – | – | 0.059 | 0.020 | 0.013 | – | – | – |
| OH | 0.043 | 0.029 | 0.176 | 0.394 | – | – | **0.089** | 0.049 | 0.013 | 0.122 | – | – |
| NY | 0.108 | 0.094 | 0.111 | **0.628** | 0.065 | – | 0.064 | 0.026 | **0.007** | 0.061 | 0.021 | – |
|  | **Curvature** | | | | | | **Wiggle** | | | | | |
| AL | – | – | – | – | – | – | – | – | – | – | – | – |
| NC | 0.047 | – | – | – | – | – | 0.053 | – | – | – | – | – |
| KY | 0.013 | 0.055 | – | – | – | – | 0.011 | 0.048 | – | – | – | – |
| IN | 0.020 | 0.026 | 0.031 | – | – | – | 0.023 | 0.023 | 0.035 | – | – | – |
| OH | 0.027 | **0.074** | **0.012** | 0.031 | – | – | 0.024 | 0.064 | **0.010** | 0.027 | – | – |
| NY | 0.024 | 0.020 | 0.034 | 0.050 | 0.050 | – | 0.027 | 0.018 | 0.038 | 0.044 | **0.056** | – |

|  | **Total** | | | | | |
| --- | --- | --- | --- | --- | --- | --- |
|  | AL | NC | KY | IN | OH | NY |
| AL | – | – | – | – | – | – |
| NC | 0.370 | – | – | – | – | – |
| KY | 0.495 | 0.684 | – | – | – | – |
| IN | 0.424 | **0.203** | 0.646 | – | – | – |
| OH | 0.255 | 0.465 | 0.270 | 0.647 | – | – |
| NY | 0.426 | 0.216 | 0.482 | **0.780** | 0.312 | – |

**Table S1b) Lifespan**

|  | **Offset** | | | | | | **Slope** | | | | | |
| --- | --- | --- | --- | --- | --- | --- | --- | --- | --- | --- | --- | --- |
|  | AL | NC | KY | IN | OH | NY | AL | NC | KY | IN | OH | NY |
| AL | – | – | – | – | – | – | – | – | – | – | – | – |
| NC | 0.066 | – | – | – | – | – | 0.065 | – | – | – | – | – |
| KY | 0.330 | 0.394 | – | – | – | – | 0.132 | 0.082 | – | – | – | – |
| IN | 0.037 | 0.103 | 0.294 | – | – | – | 0.083 | 0.021 | 0.061 | – | – | – |
| OH | 0.093 | 0.158 | 0.240 | 0.376 | – | – | 0.155 | 0.098 | **0.003** | **0.157** | – | – |
| NY | 0.123 | 0.188 | 0.210 | **0.608** | **0.030** | – | 0.085 | 0.027 | 0.054 | 0.033 | 0.064 | – |
|  | **Curvature** | | | | | | **Wiggle** | | | | | |
| AL | – | – | – | – | – | – | – | – | – | – | – | – |
| NC | 0.113 | – | – | – | – | – | 0.127 | – | – | – | – | – |
| KY | 0.017 | 0.111 | – | – | – | – | 0.015 | 0.097 | – | – | – | – |
| IN | **0.143** | 0.037 | 0.137 | – | – | – | **0.161** | 0.042 | 0.154 | – | – | – |
| OH | 0.004 | 0.111 | 0.013 | 0.061 | – | – | 0.004 | 0.098 | 0.014 | 0.053 | – | – |
| NY | 0.103 | **0.001** | 0.103 | 0.074 | 0.102 | – | 0.116 | **0.001** | 0.116 | 0.065 | 0.115 | – |

**Table S1c) Relative reduction in photosynthetic area**

|  | **Offset** | | | | | | **Slope** | | | | | |
| --- | --- | --- | --- | --- | --- | --- | --- | --- | --- | --- | --- | --- |
|  | AL | NC | KY | IN | OH | NY | AL | NC | KY | IN | OH | NY |
| AL | – | – | – | – | – | – | – | – | – | – | – | – |
| NC | **-0.016** | – | – | – | – | – | **-0.003** | – | – | – | – | – |
| KY | -0.207 | -0.191 | – | – | – | – | -0.039 | -0.043 | – | – | – | – |
| IN | -0.037 | -0.021 | -0.170 | – | – | – | -0.017 | -0.014 | -0.059 | – | – | – |
| OH | -0.064 | -0.048 | -0.143 | -0.264 | – | – | -0.005 | -0.002 | -0.046 | -0.073 | – | – |
| NY | -0.044 | -0.028 | -0.082 | **-0.740** | -0.020 | – | -0.015 | -0.018 | -0.012 | **-0.133** | -0.021 | – |
|  | **Curvature** | | | | | | **Wiggle** | | | | | |
| AL | – | – | – | – | – | – | – | – | – | – | – | – |
| NC | -0.072 | – | – | – | – | – | -0.081 | – | – | – | – | – |
| KY | -0.009 | **-0.089** | – | – | – | – | -0.008 | -0.078 | – | – | – | – |
| IN | -0.070 | **-0.003** | -0.087 | – | – | – | -0.079 | **-0.002** | **-0.098** | – | – | – |
| OH | -0.014 | -0.060 | -0.025 | -0.062 | – | – | -0.016 | -0.053 | -0.028 | -0.054 | – | – |
| NY | -0.028 | -0.046 | -0.020 | -0.005 | -0.015 | – | -0.031 | -0.040 | -0.023 | -0.004 | -0.016 | – |

|  | **Total** | | | | | |
| --- | --- | --- | --- | --- | --- | --- |
|  | AL | NC | KY | IN | OH | NY |
| AL | – | – | – | – | – | – |
| NC | -0.172 | – | – | – | – | – |
| KY | -0.264 | -0.401 | – | – | – | – |
| IN | -0.204 | **-0.041** | -0.414 | – | – | – |
| OH | -0.098 | -0.163 | -0.242 | -0.454 | – | – |
| NY | -0.119 | -0.132 | -0.136 | **-0.882** | -0.071 | – |
